# A LiDAR and IMU Integrated Indoor Navigation System for UAVs and Its Application in Real-Time Pipeline Classification

**DOI:** 10.3390/s17061268

**Published:** 2017-06-02

**Authors:** G. Ajay Kumar, Ashok Kumar Patil, Rekha Patil, Seong Sill Park, Young Ho Chai

**Affiliations:** Graduate School of Advanced Imaging Science, Multimedia and Film Chung-Ang University, Seoul 156-756, Korea; ajay@cau.ac.kr (G.A.K.); ashokpatil03@hotmail.com (A.K.P.); rekha.patil@outlook.com (R.P.); pssil1321@gmail.com (S.S.P.)

**Keywords:** scan matching, indoor navigation, indoor mapping, indoor UAV tracking, 3D model reconstruction, pipeline, classification

## Abstract

Mapping the environment of a vehicle and localizing a vehicle within that unknown environment are complex issues. Although many approaches based on various types of sensory inputs and computational concepts have been successfully utilized for ground robot localization, there is difficulty in localizing an unmanned aerial vehicle (UAV) due to variation in altitude and motion dynamics. This paper proposes a robust and efficient indoor mapping and localization solution for a UAV integrated with low-cost Light Detection and Ranging (LiDAR) and Inertial Measurement Unit (IMU) sensors. Considering the advantage of the typical geometric structure of indoor environments, the planar position of UAVs can be efficiently calculated from a point-to-point scan matching algorithm using measurements from a horizontally scanning primary LiDAR. The altitude of the UAV with respect to the floor can be estimated accurately using a vertically scanning secondary LiDAR scanner, which is mounted orthogonally to the primary LiDAR. Furthermore, a Kalman filter is used to derive the 3D position by fusing primary and secondary LiDAR data. Additionally, this work presents a novel method for its application in the real-time classification of a pipeline in an indoor map by integrating the proposed navigation approach. Classification of the pipeline is based on the pipe radius estimation considering the region of interest (ROI) and the typical angle. The ROI is selected by finding the nearest neighbors of the selected seed point in the pipeline point cloud, and the typical angle is estimated with the directional histogram. Experimental results are provided to determine the feasibility of the proposed navigation system and its integration with real-time application in industrial plant engineering.

## 1. Introduction

There is a growing need for autonomous robots in applications such as rescue, disaster assessment, plant engineering, or other tasks that would be a risky or impossible for a human to perform that requires efficient localization techniques for robots. The problem of localizing autonomous robots in unknown environments is typically solved using a low-cost scanning laser or another type of range finder; this approach has seen successful for large- to medium-sized ground robots [1,2]. Unfortunately, localization of unmanned aerial vehicles (UAVs) in an indoor environment suffers from high computational costs and the need for a number of sensory inputs. Although the outdoor positioning of UAVs has greatly benefited from the advancement of global positioning system (GPS) and inertial navigation system (INS) measurements, GPS-based localization remains impractical in closed environments due to high attenuation of the GPS signal in an indoor environment.

Recently, numerous works have appeared to solve the problem of localizing UAVs in an indoor environment. The most popular solution for localizing UAVs is the Simultaneous Localization and Mapping (SLAM) technique, which is widely applied in robotics. Compared with vision-based SLAM systems, laser-based systems provide more accurate indoor maps and models [3]. Based on laser scanners, many SLAM systems have been proposed for ground robots [4,5] and UAV platforms [6,7]. However, several of these systems are suitable only for a partially known confined indoor environment, and some suffer from computational complexity and inaccuracy.

Generally, optical range finders perform 2D laser scanning of vehicle surroundings and provide data with high resolution and at high sampling rates. Processing of such data is computationally expensive and requires massive computing resources [8]. In contrast, the control systems of UAV platforms are usually embedded devices with limited resources, low performance, and a very small amount of memory. Moreover, due to size constraints, payload capabilities are also diminished, and the UAV can only carry minimal amounts of payload in order to achieve the mission objectives. Therefore, there is a clear need for innovative laser scan processing and a scan matching method with a good trade-off between accuracy and computational complexity. Hence, it is preferable to use a point-to-point scan matching algorithm that is accurate and computationally efficient for UAV’s platform instead of Iterative Closet Point (ICP) [9], Cox [10], Complete Line Segment (CLS) [11], Normal Distributions Transform (NDT) [12], or Perimeter-Based Polar Scan Matching [13].

In this paper, a point-to-point scan matching method is used to solve the indoor navigation problem of UAVs. This method does not require the environment to be structured or contain pre-defined features and is much faster than other traditional methods. Moreover, the UAV localization algorithm was designed so that an Intel NUC-based embedded on-board computer is sufficient for computational needs. Further, the altitude of the UAV with respect to the indoor floor is calculated using line segment extraction [6] with a *split-and-merge* segmentation algorithm [14].

In addition, a novel approach is presented for the real-time classification of a pipeline in the generated indoor map by integrating the proposed SLAM method. The generated indoor map resulting from the proposed method can be utilized for visualization of the 3D environment, extraction and classification of object information [15], or for the reconstruction of as-built 3D modelling applications [16,17]. 3D data is becoming progressively more popular in reverse engineering. In particular, point clouds are one of the most primitive and fundamental representations of 3D objects acquired from LiDAR, and their data has a unique characteristic representation for automatic identification and classification of 3D objects. Thus, working directly with such representation is critical, although it is extremely challenging. The presented system has potential for use in UAV applications for real-time classification of pipelines in indoor environments.

**Figure 1:**
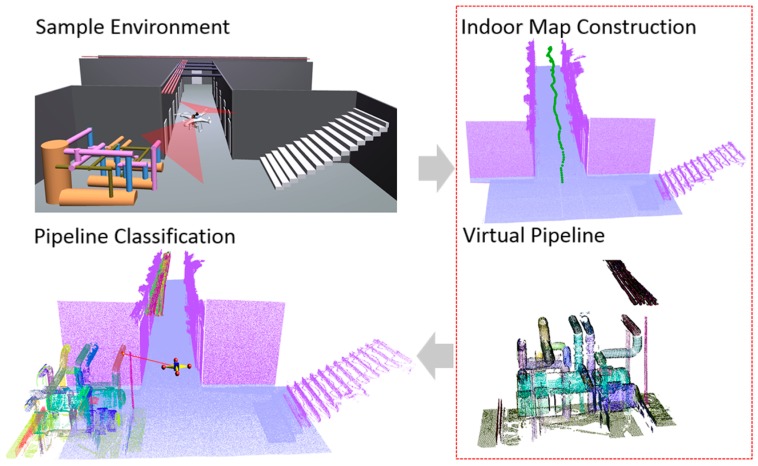
Indoor navigation and mapping overview.

Finally, we show that the proposed SLAM system can be used in indoor mapping and modelling applications, as illustrated in Figure 1. The contributions of the work presented may be summarized as follows:

(1) The use of a modified PSM algorithm in orientation estimation and selecting the ideal plane for the scan matching algorithm with application to robust 3D mapping.
(2) Presentation of a novel approach for real-time pipeline classification in the generated indoor map using a histogram-based radius estimation.

## 2. Related Work

In this section, different scan matching-based SLAM approaches are presented along with a LiDAR and IMU integrated navigation system for indoor UAVs. Also, this section presents a different pipeline classification method for application in plant engineering.

### 2.1. Scan Matching Methods for SLAM

Iterative Closest Point (ICP) is an iterative algorithm that aligns two consecutive laser scans without extracting the features, such as lines and planes. Since it does not extract features, ICP is applicable even for unstructured environments where such features do not exist. It is however, slower than other methods because it uses the whole point cloud to find an optimal transformation. A SLAM method based on stereo vision and the ICP algorithm has been described in [18]. A SLAM method based on laser scan matching has been introduced in [19]. Besl and McKay [20] proposed an ICP in which, for each point of a current scan, the point with the smallest Euclidean distance in the reference scan is selected. There are other variants of ICP algorithms proposed in [21,22] that are used in laser scan matching. However, the performance of ICPs is not stable since the algorithm involves an iterative process, i.e., it tries to improve the matching until convergence. ICPs fail to converge to a correct solution when a good initial guess of the transformation is unavailable.

The probabilistic scan matching (Probabilistic SM) method proposed in [23] is like classical ICP and consists of two stages, i.e., correspondence location and transformation estimation. In the first stage, correspondence between the scans is computed; next, relative displacement is estimated. The probabilistic model of this process takes into account all of the uncertainties involved with displacement of the sensor and the measurement noise. In addition, Probabilistic SM allows correspondence to be found through probability integration over all possible associations between the range measurements of the two scans. However, this probabilistic modeling of uncertainty is more suitable to real sensor data compared with pure geometrical approaches.

The feature-based 2D laser scanning proposed in [3] estimates the relative positions and orientation of two laser scans by matching point and line features extracted from each laser scan. Feature points are detected based on an extended 1D SIFT, and line features are extracted using a split-merge algorithm. Orientation information is extracted from 2D line correspondence, which helps to reject feature point outliers and provides a good initial guess for transformation estimation. Also, the method includes an effective strategy to discard unreliable features. Although the method is more accurate and robust than ICP, it requires high computational resources in order to process each scan in real-time. Using this approach, it is very difficult to achieve accuracy with the diminished resources of the UAV platform.

### 2.2. Indoor UAV Navigation

A comprehensive UAV indoor navigation system proposed by Wang and Cui in [24] is based on the vision optical flow and Laser FastSLAM. In this system, the UAV is embedded with three main sensors, an IMU, a camera, and a laser scanner. The planar position of the UAV is estimated from the FastSLAM scan matching algorithm. In FastSLAM algorithms, corners and line segments are considered map features to perform SLAM. Along with this, UAV height is captured from the barometer. Even though the UAV path is well estimated with minimum hardware resources, mapping of the environment is performed offline, and the computational complexity needs to be reduced.

Fei and Kangli [6] proposed a robust and efficient navigation solution for a UAV for an indoor environment. The main sensors used on-board the UAV are two scanning laser range finders and an inertial measurement unit. The plane position of the UAV is efficiently calculated from the laser scanner mounted horizontally. The height of the UAV with respect to the floor is calculated directly from the vertically mounted laser scanner. Here, the authors used the *split-and-merge* segmentation algorithm [14] to extract the line segments from the LiDAR measurements. This navigation solution uses a planar localization algorithm with two main assumptions. Based on the planar localization algorithm, translation and rotational variations are estimated efficiently. This planar motion algorithm includes four steps: feature extraction, rotation tracking, corner feature association, and position tracking. However, this navigation solution is limited to a partially known environment.

Another UAV localization method for indoor environments based on a LiDAR sensor, entitled “Autonomous flight in unknown indoor environments” [25] and proposed by A Bachrach and R He, uses a single LiDAR sensor for localization and map construction. For planar localization, the authors used a high-speed laser scan matching algorithm based on the globally consistent range image alignment method. But, the approach has limited field of view when applied to environment mapping rather than to navigation. However, UAV applications such as mapping and real time object classification in a generated map require the acquisition of sufficient geometric information. The described platform setup is used to generate 3D maps only when the UAV moves in a vertical direction, as it is too difficult to acquire geometric information of objects such as spheres and cylinders in a horizontal direction. Also, the method relies only on a small field of LiDAR data to estimate the altitude; thus, it can be very difficult to distinguish between an actual floor surface and other objects on the floor.

The LiDAR/IMU integrated navigation solution proposed by Liu and Shifei [5] is a type of autonomous integrated technology. LiDAR and IMU measurements are used to drive the simplified strap down INS equations. In the first step, the method extracts features from the LiDAR measurements. Next, MEMS INS measurements are used to compensate for errors in the LiDAR measurements during altitude maneuvering of the UAV. Finally, a Kalman filter is used to combine the measurements from LiDAR and MEMS IMU to estimate the relative position. Since the matching algorithm depends on the line features of the environment, it is difficult to apply this method in a featureless environment.

### 2.3. Applications in Plant Engineering

There has been growing demand for the 3D object classification and reconstruction of as-built models from point cloud data. Current object classification methods generally fall into two categories. First, real-time object classification continues to be an active area of research [16]. Methods belonging to the second category operate offline with automated classification of specific objects [15,17].

A study by Czerniawski et al. [16] presented an estimation of pipe radius in cluttered point cloud data. The algorithm is used for estimating pipe radius in point clouds generated by a 3D sensor. Initially, a random point is selected from the input point cloud, and its nearest neighborhood is isolated as a subset. Then, a rough estimate of the surface normal is calculated using neighbor points based on Principal Component Analysis (PCA). The computed surface normal will act as the axis of rotation for the planes passing through a selected seed point with uniform rotation around the surface normal. The points sufficiently close to the plane are projected onto the plane that fits a 2D circle, and a minimum radius of the fitted circle is identified and assumed to be associated with the radius of the pipe. This method operates poorly when the subset of points is minimized.

Kim et al. [17] use prior knowledge of 3D CAD models for classification of objects, which are available in Piping and Instrumentation Diagrams (P&ID), and match topography with the acquired point cloud data. The method has a high rate of recall compared to recent research, but it requires a huge database and is complicated to use in real-time classification. Huang et al. [15] proposed a per-point classification method by combining Support Vector Machine (SVM) and Fast Point Feature Histograms (FPFH). SVM is one of the supervised approaches for point cloud classification. However, SVM-based classification methods require prior object labeling and matching in a 3D environment and involve a time-consuming process that makes real-time classification inefficient.

## 3. LiDAR and IMU Integrated Navigation

In this section, a LiDAR and IMU integrated navigation system is proposed. This UAV navigation system includes an existing 2D laser scan matching method for planar localization of UAVs and altitude estimation using a low range LiDAR sensor. Also, modifications made to the scan matching process to overcome altitude angle variation during the flight of a UAV are described in detail.

### 3.1. Point-to-Point Scan Matching

Planar localization of a UAV is achieved using measurements from the horizontally scanning primary LiDAR sensor mounted on the on-board computer. Scan measurements from the LiDAR scanner can be understood as a momentary snapshot of the surrounding environment of the UAV. The point-to-point matching-based Polar Scan Matching (PSM) method [19] is used to determine the relative translation of consecutive LiDAR scans during the flight of the UAV. Each scan covers 270° of the surrounding environment of the UAV, with 0.25° of angular resolution. The point in each measured angle indicates the distance between the UAV and the nearest obstacle in the corresponding direction. This approach is best suited for use with portable embedded devices because of its low computational requirements and high accuracy.

As mentioned before, we follow the point-to-point matching based on the PSM approach proposed by Diosi and Kleeman [19] in order to determine the relative translation. In this matching method, the current scan is aligned with respect to the reference scan so that the sum of the square range residuals is minimized.

The current scan is described as $S=(x_{c},y_{c},\theta_{c},{\left\{r_{ci},\varphi_{ci}\right\}}_{i=1}^{n})$, where $x_{c},y_{c},\theta_{c}$ describes position and orientation, and ${\left\{r_{ci},\varphi_{ci}\right\}}_{i=1}^{n}$ describes *n* range measurements $r_{ci}$ at bearings $\varphi_{ci}$, expressed in the current scan coordinate system. The reference scan is described as $R={\left\{r_{ri},\varphi_{ri}\right\}}_{i=1}^{n}$.

The scan matching process works as follows: scan pre-processing and scan projection are followed by translation estimation, which is alternated with scan projection and followed by orientation estimation.

Prior to matching, spurious measurements and objects that are likely to move are removed from the scan data using a median filter and a scan segmentation filter in order to increase the accuracy and robustness of scan matching. Next, the current scan acquired at position *S* is projected with respect to the reference scan acquired at position *R* such that it reflects how the current scan would look if it were performed from the reference position *R*. The range bearings of points from position *R* are calculated using Equations (1) and (2):

(1) $$r_{ci}^{\prime }=\sqrt{{\left(r_{ci}\cos(\theta_{c}+\phi_{ci})+x_{c}\right)}^{2}+{\left(r_{ci}\sin(\theta_{c}+\phi_{ci})+y_{c}\right)}^{2}}$$

(2) $$\phi_{ci}^{\prime }=\tan^{-1}2(r_{ci}\sin(\theta_{c}+\phi_{ci})+y_{c},r_{ci}\cos(\theta_{c}+\phi_{ci})+x_{c})$$

**Figure 2:**
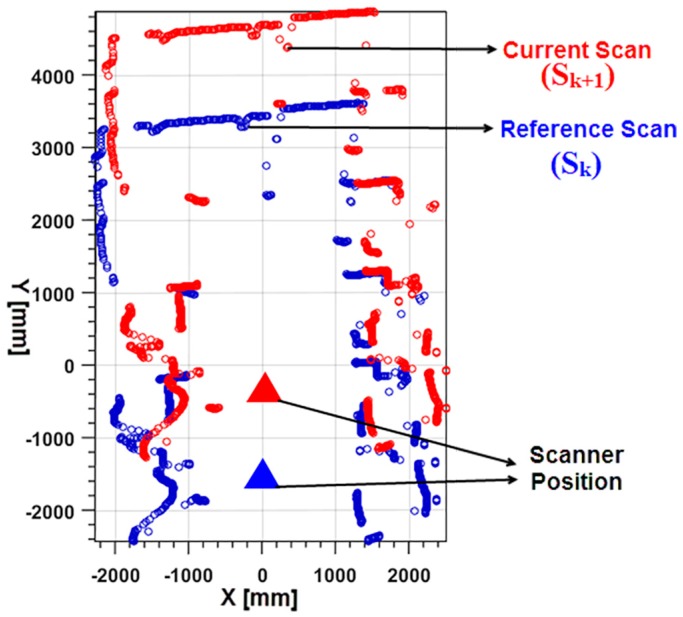
Projected reference and current scan.

Here, $\tan^{-1}$ is the four-quadrant version of arctan, and ($r_{ci}^{\prime }$, $\phi_{ci}^{\prime }$) represents the projected current scan readings. Next, the aim is to estimate what the laser scanner would measure from reference position *R*. The re-sampled range value for each sample bearing using linear interpolation is represented by the notation ($r_{ci}^{\prime \prime }$, $\phi_{ci}^{\prime \prime }$). Since the association rule is to match the bearings of the points, the next ranges $r_{ci}^{\prime \prime }$ at the reference scan bearings $\phi_{ci}^{\prime \prime }$ are calculated using interpolation. Figure 2 shows the projection of consecutive scan points based on the rule mentioned above.

#### 3.1.1. Orientation Estimation

**Figure 3:**
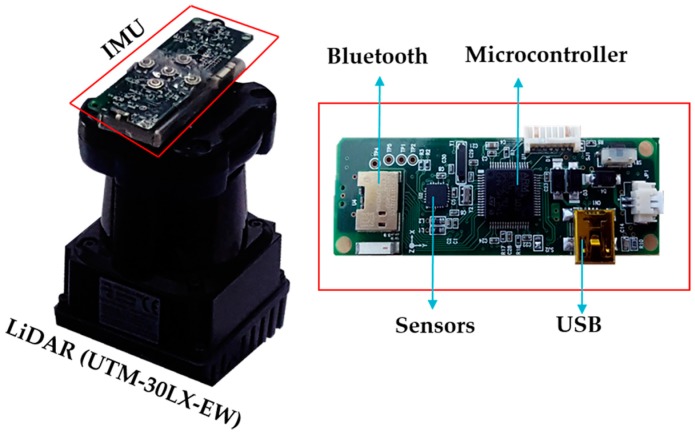
IMU integrated LiDAR.

Orientation of the current scan is obtained from a wireless IMU integrated with a LiDAR, as shown in Figure 3. The custom-made wireless IMU includes a tri-axis gyroscope, tri-axis accelerometer, and tri-axis magnetometer. All of these sensors are connected to a microcontroller that collects and processes the data. A complementary filter sensor fusion algorithm [26] with drift compensation is implemented to obtain precise and accurate 3D attitude data [27]. The quaternion complementary provides a normalized quaternion output from the calibrated sensor data as input.

The normalized quaternion output *Q* from IMU is a vector with four elements [*q*_1_ *q*_2_ *q*_3_ *q*_4_], from which Euler angles are derived using Equations (7)–(9) in ZYX sequence, and the current scan measurement $S={\left\{r_{i},\varphi_{i}\right\}}_{i=1}^{n}$ is obtained from the LiDAR.

Let *t* and *t* + 1 be the two successive time instants at which two LiDAR scans are acquired, and let P(t) and P(t+1) be the corresponding point clouds generated from the scan measurements with integrated IMU measurements that can be described as shown in Equation (3):

(3) $$P(t)=((x_{t},y_{t},\theta_{t}),{\left\{r_{i},\varphi_{i}\right\}}_{i=1}^{n})\;\mathrm{and}\;P(t+1)=((x_{t+1},y_{t+1},\theta_{t+1}),{\left\{r_{i},\varphi_{i}\right\}}_{i=1}^{n})$$

where $\left(x_{t},y_{t},\theta_{t\;}\right)$ and $\left(x_{t+1},y_{t+1},\theta_{t+1\;}\right)$ represent the pose of the scanner at reference and current locations, respectively. ${\left\{r_{i},\varphi_{i}\right\}}_{i=1}^{n}$ is the range measurement obtained.

Using range measurements ${\left\{r_{i},\varphi_{i}\right\}}_{i=1}^{n}$, the coordinate point is estimated using Equation (4) and stored in the point cloud $p_{t}$ and $p_{t+1}$, respectively, at the tth and t+1th time stamps:

(4) $$\left[\begin{array}{c} x_{i}\\ y_{i}\\ z_{i} \end{array}\right]\;=\;\left[\begin{array}{c} r_{i}\;\cdot \;\cos\left(\varphi_{i}-\frac{3\pi }{4}\right)\\ 0\\ r_{i}\;\cdot \;\sin\left(\varphi_{i}-\frac{3\pi }{4}\right) \end{array}\right]$$

Then pose of the LiDAR from the reference and current scanner locations can be represented using the matrix form in Equation (5):

(5) $$T_{t}\;=\;\left[\begin{array}{ccc} \cos\;\theta_{t} & -\sin\;\theta_{t} & x_{t}\\ \sin\;\theta_{t} & \cos\;\theta_{t} & y_{t}\\ 0 & 0 & 1 \end{array}\right]\;\mathrm{and}\;T_{t+1}\;=\;\left[\begin{array}{ccc} \cos\;\theta_{t+1} & -\sin\;\theta_{t+1} & x_{t+1}\\ \sin\;\theta_{t+1} & \cos\;\theta_{t+1} & y_{t+1}\\ 0 & 0 & 1 \end{array}\right],$$

where $\theta_{t}$ and $\theta_{t+1}$ are the Euler angle’s computed from the quaternion $Q_{t}$ and $Q_{t+1}$ respectively, using Equation (9). $T_{t}$ and $T_{t+1}\;$ are the homogeneous transformation matrix. Finally transformed point cloud scans obtained by multiplying the transformation and point cloud matrix as shown in the Equation (6):

(6) $$P(t)\;=\;T_{t}\;p_{t}\;\mathrm{and}\;P(t+1)\;=\;T_{t+1}\;p_{t+1}$$

Where $T_{t}$ and $T_{t+1}\;$ are the transformation matrices, $p_{t}$ and $p_{t+1}$ are the point clouds obtained at the tth and t+1th time stamps, respectively.

Now, after projecting the current scan line with respect to the reference scan, the angular difference $\theta_{c}$ between the reference and current scan can determined as:

(7) $$\theta_{c}\;=\;\theta_{t+1}\;-\;\theta_{t}$$

**Figure 4:**
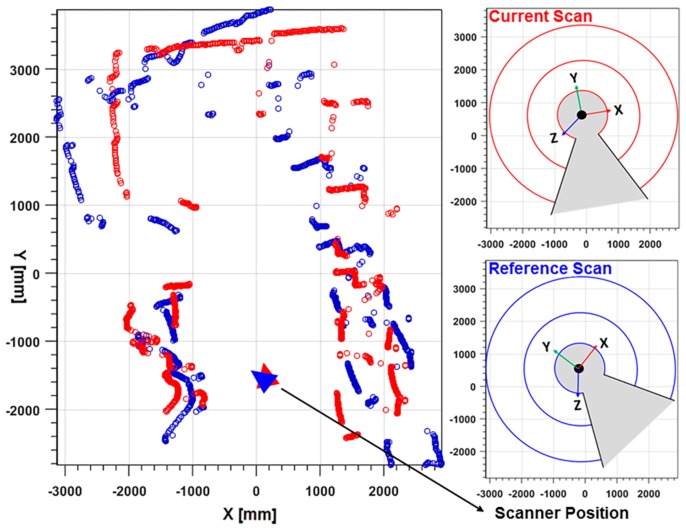
Projected scan points according to scanner orientation using IMU.

Figure 4 shows the projected two consecutive scans according to the orientation of the scanner obtained from the integrated IMU. In the figure, the reference scan and currents scan points are represented in blue and red colored circles, respectively. The right side of the image presents the scanner Field of View (FOV) with respect to orientation.

#### 3.1.2. Ideal Scanner Plane

The primary LiDAR mounted on the on-board computer on a UAV detects obstacle information in the 2D scanning plane. In the case of ground robots, which always move in a plane, the altitude angle is maintained at an almost constant level. However, it is very difficult to control a UAV at a constant altitude angle during flight in the indoor environment. Therefore, LiDAR measurement information from the UAV platform cannot accurately describe the obstacle distribution in the horizontal plane.

This leads to an enormous amount of error in the scan matching process during relative position estimation. Therefore, it is necessary to reduce the influence of altitude angle variation during LiDAR measurement.

**Figure 5:**
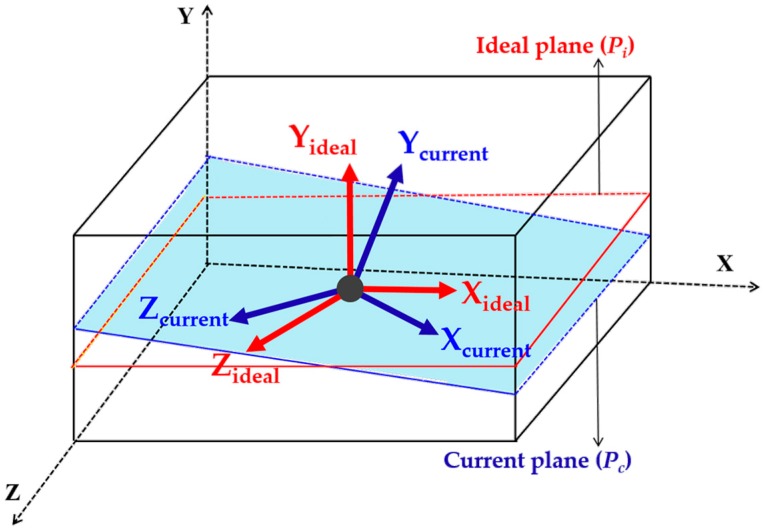
Ideal plane detection for scan matching.

As shown in Figure 5, the actual measurement plane $P_{i}$ of LiDAR is defined by the coordinates $P_{i}(x_{i},y_{i},z_{i})$, and $P_{c}(x_{c},y_{c},z_{c})$ are the coordinates defining the measurement plane $P_{c}$ after variation in the altitude angle of the UAV.

The angular variation occurring during flight of the UAV is captured from the roll and pitch angles of the 6DOF IMU integrated with the primary LiDAR. Quaternion output from the IMU sensor are converted to Euler angles using Equations (8)–(10), which use the ZYX sequence.

Considering a unit quaternion ‖q‖, the norm $\left\|q\right\|=q_{1}^{2}+q_{2}^{2}+q_{3}^{2}+q_{4}^{2}=1$

(8) $$\phi =\tan^{-1}2(2q_{3}q_{4}-2q_{1}q_{2},\;2q_{1}^{2}-1+2q_{4}^{2})$$

(9) $$\theta =-\sin^{-1}(2q_{2}q_{4}+2q_{1}q_{3})$$

(10) $$\psi =\tan^{-1}2(2q_{2}q_{3}-2q_{1}q_{4},\;2q_{1}^{2}-1+2q_{2}^{2})$$

where ϕ (roll) represents the rotation around the Z-axis, θ (pitch) represents the rotation around the Y-axis, and ψ (yaw) represents the rotation around the X-axis.

**Figure 6:**
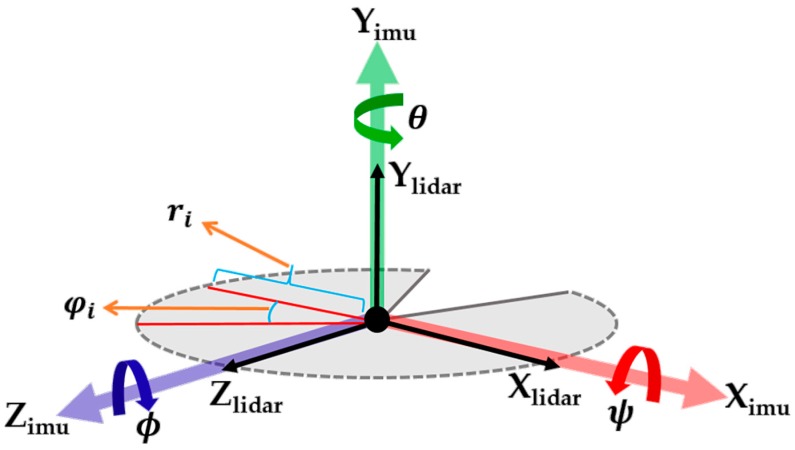
LiDAR and IMU coordinate frames.

Each scan measurement from the LiDAR is associated with the ϕ (roll) and ψ (yaw) angles acquired using integrated IMU as shown in Figure 6. Before performing scan matching between the two consecutive scan lines, the vertical angular difference between the scan lines is computed. Depending on the pre-defined threshold, scan lines are considered for the matching process; otherwise, scan lines will be rejected. Formulation of the selection of valid plane measurements for the scan matching process is as follows.

Given two 2D laser scans, $S_{t}$ is calculated from relative position t, and $S_{t+1}$ is relative to positions t+1 along with $\left(\phi_{t},\psi_{t}\right)$ and $\left(\phi_{t+1},\psi_{t+1}\right)$ angles, respectively:

(11) $$S_{t+1}=\left\{ \begin{array}{l} s_{t+1}^{temp}\;(\phi_{t+1}-\phi_{t})<\phi_{threshold}\;\&\;(\psi_{t+1}-\psi_{t})<\psi_{threshold}\\ invalid \end{array}\right.$$

In the case of invalid plane measurement, the current scan $S_{t+1}^{temp}$ is rejected, and the next scan that satisfies Equation (11) will be considered for further matching. In the above equation, $\phi_{threshold}\;=\;2.6{}^{\circ}$ and $\psi_{threshold}\;=2.4{}^{\circ}$ are estimated based on the average tilting angle observed in multiple flights of UAV in different indoor environments such as a long corridor and a closed room. During the test flight of a UAV, we obtained 1.14° as minimum and 4.23° as maximum angular differences in consecutive time steps in roll angles and 1.28° minimum and 3.64° maximum yaw angles. Based on these observations, we considered the average angle as the threshold to identify the valid plane for scan matching.

### 3.2. UAV Altitude Estimation

UAV altitude measurement in an indoor environment with a completely flat floor can be obtained directly from the low range finder. However, there are some cases where altitude estimation fails because of disturbances from objects such as tables, chairs, or windowsills in an indoor environment. In order to solve this problem, a floor detection algorithm [6] is used with a secondary laser scanner mounted orthogonally to the primary scanner.

Using a split-and-merge line extraction algorithm [14], measurements from the secondary LiDAR data can be filtered by line segments. These line segments are sorted by perpendicular distance between the ground and the laser scanner center. The furthest line segments are retained, and among them, the longest one is believed to be true ground. Finally, the UAV height can be obtained easily as the perpendicular distance of this line to the laser scanner center. Using this method, an accurate height measurement can be obtained as long as the laser scanner projects a portion of its laser beams onto the true ground.

### 3.3. Kalman Filtering

**Figure 7:**
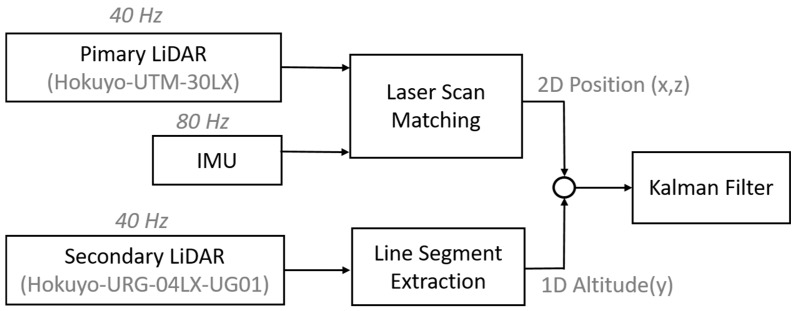
Fusion of 2D and 1D measurement data.

In order to integrate and filter the 2D position data from scan matching and 1D altitude data from line extraction, we also implemented a linear Kalman filtering algorithm, similar to the work presented in [28]. The block diagram of the integration process is shown in Figure 7. The filter includes three states shown in Equation (12), where two states are for the 2D position from the scan matching algorithm, and one state is for the altitude data from the line extraction algorithm.

(12) $$x_{k}=[posX_{k},posY_{k},posZ_{k}]$$

where $x_{k}$ is a measurement vector or current state of the system.

$posX_{k}$: *X* coordinate of the 2D position at the *k*th time interval from the scan matching algorithm.
$posY_{k}$: *Y* coordinate of the 1D position at the *k*th time interval from the altitude estimation algorithm.
$posZ_{k}$: *Z* coordinate of the 2D position at the *k*th time interval from the scan matching algorithm.

(13) $$x_{k+1}=Ax_{k}+Bu_{k}+w_{k}$$

(14) $$y_{k}=Hx_{k}+z_{k}$$

where A, B, and H are state, input, and measurement matrices, respectively, and *x* is the state of the system, *u* is a known input to the system, *y* is the measured output, *w* is the process noise, and *z* is the measurement noise. Vector *x* contains the present state of the system, which is estimated from given measurements in vector *y*. Equations (13) and (14) define the *l*inear model of the filter used [29].

**Figure 8:**
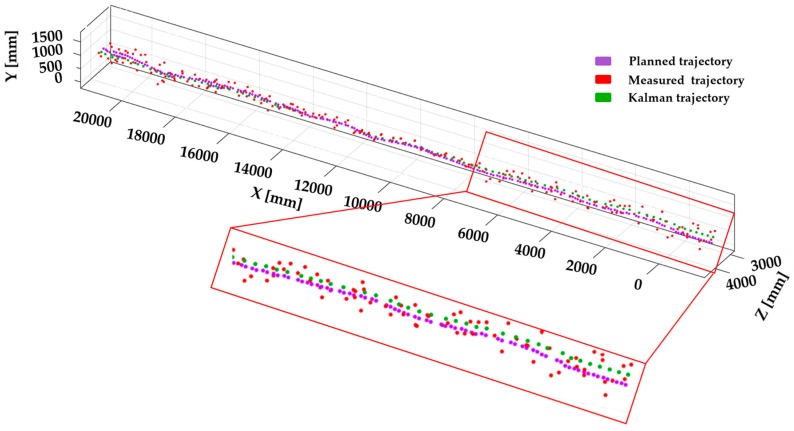
Point displacement of scan matching measurement and Kalman filtered data.

Figure 8 shows the displacement of 3D position data with respect to the planned and measured trajectory. Here, measured trajectory refers to the obtained output from the scan matching algorithm and is presented in the image with red colored dots. The planned trajectory is predefined in the known indoor corridor, where UAV is assigned with a set of commands to follow the desired trajectory and is presented with pink colored dots. Green colored dots in the image represents the Kalman filtered output of the measured trajectory. Even though the accuracy of the measured trajectory is poor compared to the planned trajectory, use of the Kalman filter increases the accuracy of the trajectory by reducing the measurement noise.

## 4. Pipeline Classification

The following sections describe the proposed pipeline classification method adopted for automatic identification of the pipe radius in real-time utilizing ray casting from a UAV position. In the first step, the ray-point cloud intersection using an octree ray casting algorithm [30] is used for interaction with the point cloud in real-time to select a seed point, and its neighboring points are calculated. In the second step, the normals of the seed point as well as its neighboring point are estimated, and the tangential vector is calculated by finding the maximum direction from the seed point. In the final step, neighboring points are paired in the direction of the tangential vector of the seed point, and the typical angle range is estimated. Depending on the typical angle and region of interest (ROI), the radius of the pipe is estimated, and pipes are classified. The following subsections describe the proposed pipeline classification method.

### 4.1. Seed-Point Selection and Its Neighboring Point Estimation

**Figure 9:**
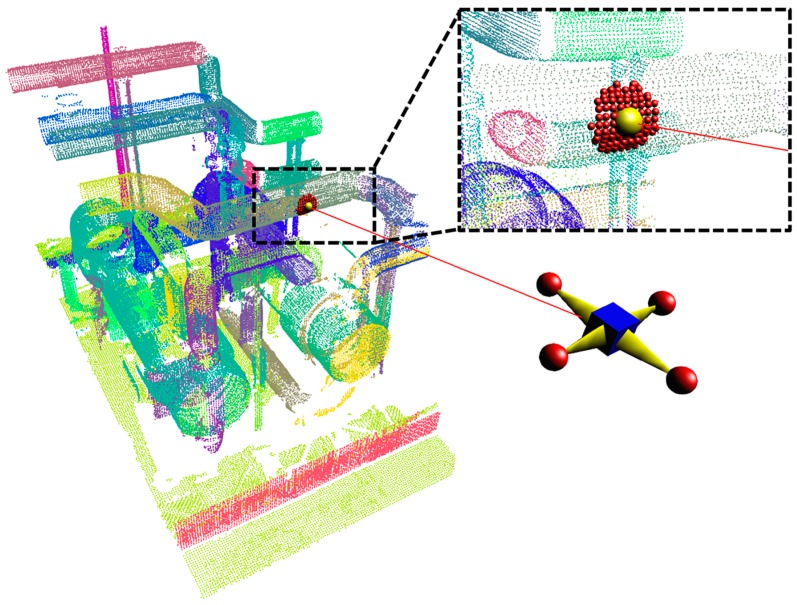
Seed point selection and its neighbor points.

The first step for pipe radius estimation in real-time is to select a seed point in the point cloud data using an octree ray-casting operation, which finds a single seed point by the ray intersection. Figure 9 shows the ray initially starting from the UAV position. If the ray hit the first voxel of the octree, the intersection of the ray will be checked with all points stored within that voxel. Among those points, the only ray intersected by the first point is obtained, and its position is returned as a seed point. The laser scanner-acquired point data are subdivided using octree voxelization by considering an appropriate voxel size of 1.0 cm. The UAV position acquired from the navigation approach is used to generate the ray to intersect the point cloud with the initial position of the UAV. A ray is represented by the line formula [31] as:

(15) $$P=R_{org}+l\cdot R_{dir}$$

where *p* is a point on the line, $R_{org}$ is the origin of the ray (i.e., UAV position), $R_{dir}$ is the direction of the ray from the UAV position, and l is the user defined length of the ray, which defines how far the end point of the ray is from the current UAV position. Next, the neighboring point of a seed point is estimated using the nearest neighbor search with the Kd-tree search algorithm [32].

A nearest neighbor algorithm will compute the distance to all points in the point cloud to the seed point. It will then sort the points in ascending order of distance and return all points whose distance is less than the defined radius (i.e., ROI). The nearest neighbor point for the seed point is estimated and shown in Figure 9.

### 4.2. Normal and Tangential Vector Estimation

**Figure 10:**
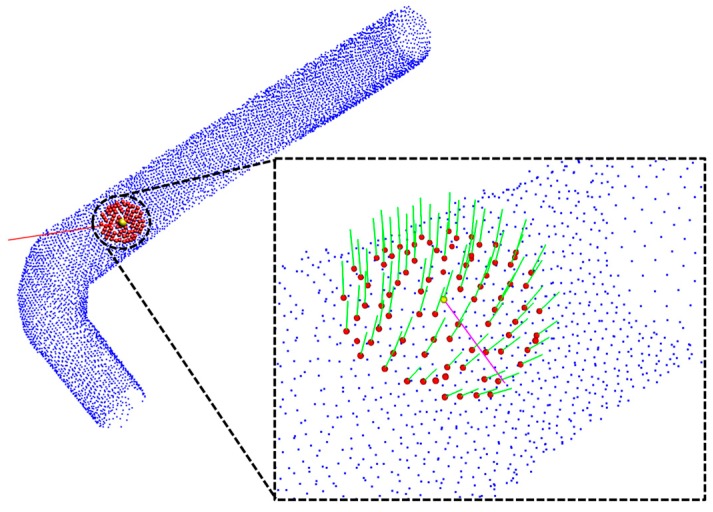
Result of normal and tangential vector estimation.

The first features extracted from a given dataset are the surface normals. For estimating the seed point normal as well as the normal of the neighboring point, the surface normal estimation method from the Point Cloud Library (PCL) is used [32]. The theoretical primer is as described below. For each point (*p_i_*), the covariance matrix is obtained as:

(16) $$c=\frac{1}{k}\sum_{i=1}^{k}(p_{i}-p)\cdot {\left(p_{i}-p\right)}^{T},c\cdot v_{j}=\lambda_{j}\cdot \vec{v_{j}},j\in \{0,1,2\},$$

where *k* is the defined number of nearest neighbors, *p* represents the centroid of the nearest neighbors, *λ_j_* is the *j*-th eigenvalue, and *v_j_* is the *j*-th eigenvector. The estimated normals are shown in Figure 10.

After estimating the normals, a tangential vector (magenta colored line shown in Figure 10) of the seed point is calculated using neighboring normals. The tangential vector is calculated by finding the maximum direction vector between the seed point to all of its neighboring points by rotating the direction vector 360°, and the maximum directional angle is the tangential vector of the seed point.

### 4.3. Point Pairing and Typical Angle Calculation

**Figure 11:**
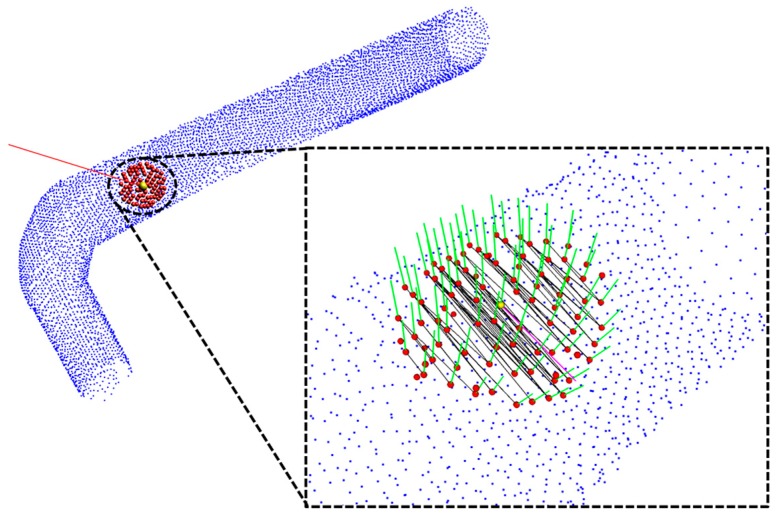
Result of point pairing using tangential vector.

After specifying the seed point direction through the tangential vector, the point pair that matches the direction of the tangent vector is found. Neighboring points are mapped to a pair matching the direction of a tangent vector specified in a pair consisting of neighboring points with a pair of N (N − 1). A matching pair is specified as a tangent vector and a pairwise direction vector. If the result of the dot product of two vectors is within a pre-determined threshold angle, it is specified as a pair matching the corresponding direction. Figure 11 shows the result of finding a pair in the specified direction by setting the threshold angle at 5°. In the figure, the black lines connect the paired points, and magenta is the direction specified through the tangent vector.

**Figure 12:**
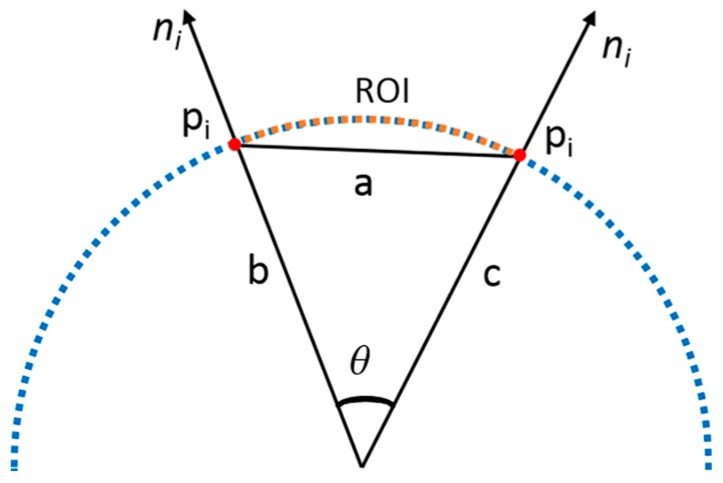
Typical angle calibration.

A typical angle is calculated for each pair of points estimated in the previous step because the angle of the maximum direction varies according to the distance of pairs within the currently selected ROI. To find the typical angle, the cosine law is utilized, as shown in Figure 12, and the formula for calculating *θ* in the figure is as follows:

(17) $$\cos\theta =\frac{b^{2}+c^{2}-a^{2}}{2bc},$$

The above Equation (15) calculates the angle *θ* using the length of the sides (a, b, and c), as shown in Figure 12.

The distance of pairs is different in the point cloud *p_i_* of the selected ROI. Therefore, the value of cos *θ* is the calculation of the dot product of surface normals of matching pairs (two points *p_i_*) by rotating the tangential vector and finding the maximum dot product result as a maximum angle. By taking a proportion of the pair distance and radius of the current region, the angle result is calibrated using the Point Feature Histogram (PFH) [33] and is stored in a bin. An average of the calibrated angles is calculated from the stored bin, and the calculated result is the typical angle. Depending on the typical angle and ROI, the radius of the cylinder is recognized and classified. Table 1 provides the pipeline classification by utilizing the typical angle range with the ROI for cylinders with different radius sizes.

**Figure 13:**
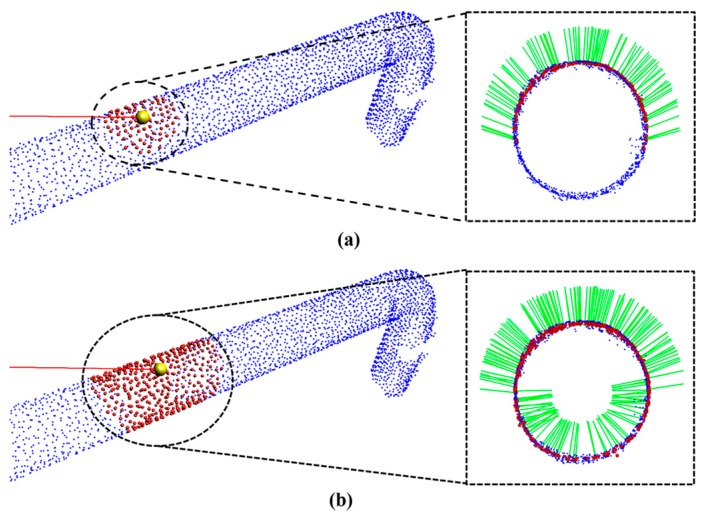
(**a**) ROI with 20cm, (**b**) ROI with 30cm.

As shown in Table 1, the range of typical angles varies according to the ROI. If the ROI is larger than 30 cm for a pipe radius of 30 cm, 25 cm, or 18 cm, then the area of the ROI is wider, and the normal estimation and the pairing of points calculation result are uncertain. The typical angle range for a 15 cm cylinder can be found with only a 10 cm or 20 cm ROI. If the ROI is set to 30 cm, then the area of the ROI is wider in the cylinder (as shown in Figure 13), and the normal estimation and the pairing of points calculation result are uncertain.

## 5. Results

### 5.1. System Overview

In order to verify the proposed UAV indoor navigation and its application in real-time pipeline classification, actual flight tests were carried out. The platform used for the work described in this paper comprises a Phantom 3 Advanced Quadcopter from the commercial UAV designer and manufacturer DJI (Shenzhen, China). This quadcopter has a maximum take-off weight of 1 kg and can complete 12~14 minutes of flight time.

**Figure 14:**
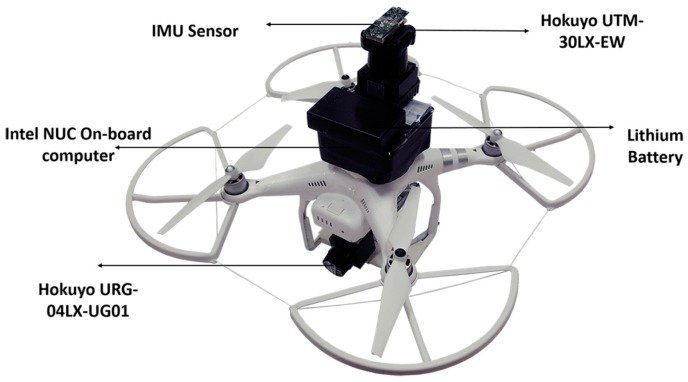
UAV equipped with LiDAR and IMU sensors.

An Intel NUC D54250WYKH i5 processor was used as the on-board computer. A UTM-30LX EWmodel of a scanning laser range finder from Hokuyo (Santa Clara, CA, USA) is used as the primary LiDAR for planar localization of the UAV, which has a 270° scanning range and 30 m coverage distance. Another URG-04LX-UG01 scanning laser range finder was used as the secondary LiDAR for the altitude estimation of the UAV. In additional to the quadcopter battery, a lithium battery with 2000 mA capacity was used to power the LiDAR and on-board computer. A 6DOF wireless inertial measurement (WIMU) device was used to acquire the UAV angular variations, which includes Magnetic, Angular Rate, and Gravity (MARG) sensors. All required computation for the localization algorithm was performed by the on-board computer, and real-time indoor map visualization along with pipeline classification related computations were performed in the host station connected through Wi-Fi communication. Figure 14 shows the UAV platform with embedded sensors.

### 5.2. Indoor Navigation and Mapping

**Figure 15:**
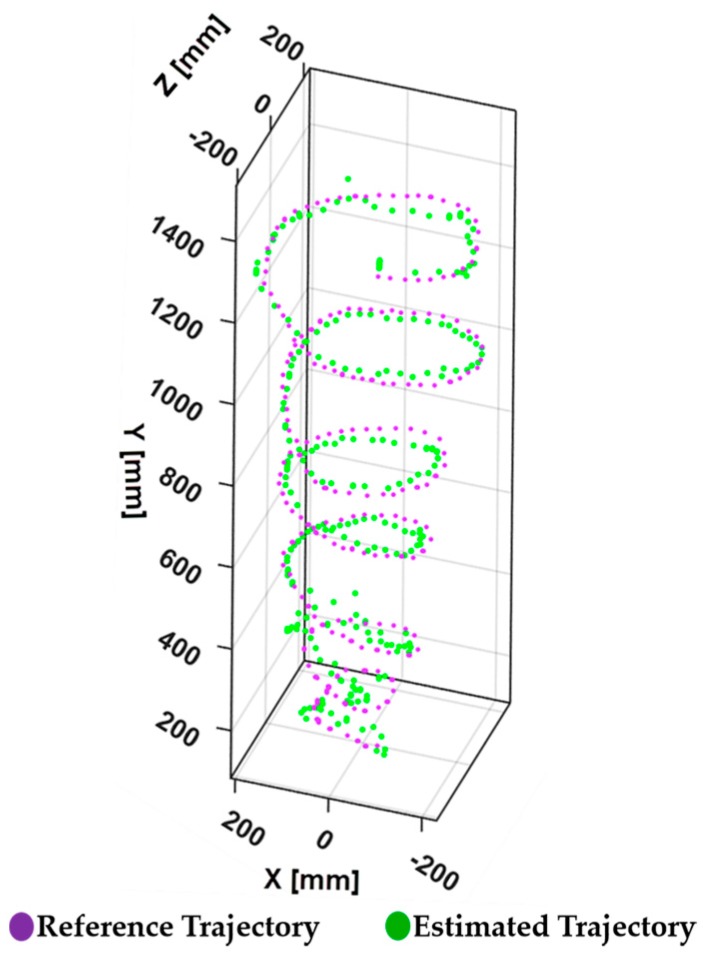
Trajectory of UAV during planned path.

**Figure 16:**
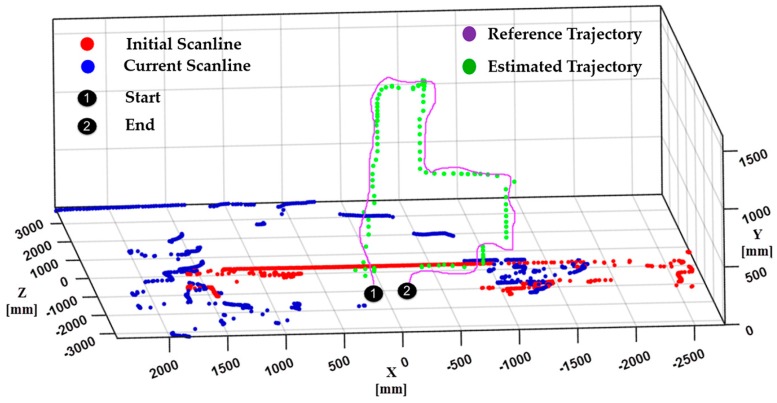
Trajectory of UAV captured during actual flight test.

The proposed navigation system results are illustrated in Figure 15 and Figure 16. Initial tests were conducted to verify the integrated performance of multiple sensors: primary LiDAR, secondary LiDAR, and IMU. In these tests, the UAV was directed to fly in different trajectories to observe the accuracy of the planar localization and altitude estimation process.

The result of the trajectory shown in Figure 15 illustrates a smooth spiral trajectory during the flight along the planned path. In this evaluation, the UAV is programmed to follow a spiral path within a radius of 2 m. The tracked spiral trajectory in Figure 15 guarantees the efficiency of the algorithm in localizing the UAV during planar motion.

Another flight test was carried out in an indoor hall in order to analyze the algorithm accuracy in terms of trajectory estimation. The experimental flight of the UAV comprises a moving drone in a reference trajectory, as shown in Figure 16, where the UAV was programmed to move up to 1 m in the Y direction and 1 m in the the X direction. Figure 16 shows that the estimated trajectory has a similar shape to the programmed reference trajectory. The overall flight test was carried out with careful control of the UAV in order to ensure stability in the corridor of the indoor environment. Specifically, during turns in the corridor, the UAV was controlled in a hold and turn fashion in order to avoid large yaw angles.

**Figure 17:**
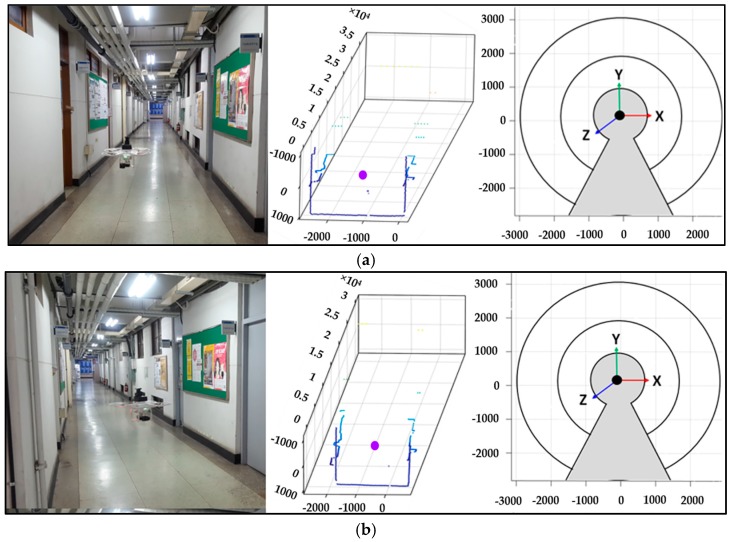

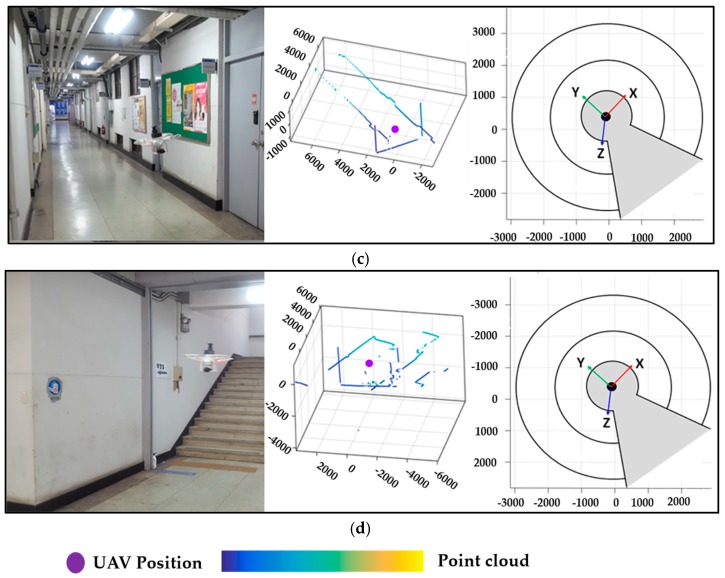
Instances of the wall and floor during flight test, (**a**) Moment 1, (**b**) Moment 2, (**c**) Moment 3, (**d**) Moment 4.

Figure 17 shows the four instances of the flight, with left column image showing the actual flying condition and the middle column illustrates the laser scanner data at that particular moment. The right column figure shows the pose of the primary LiDAR estimated using integrated IMU sensor.

**Figure 18:**
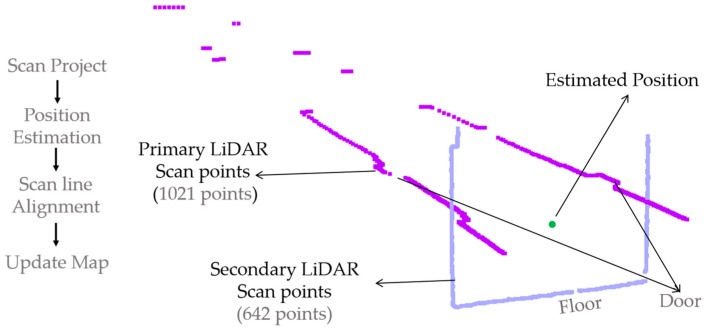
Single instance of primary and secondary LiDAR data.

Figure 18 illustrates the localization and mapping process with a single instance of scan data from the primary and secondary LiDAR. After projecting the LiDAR data, the planar UAV position is derived from the primary LiDAR using the scan matching algorithm, and the altitude of UAV is estimated using the secondary LiDAR. In order to generate the indoor map, data from both LiDAR are aligned with respect to the estimated position. As shown in Figure 18, primary LiDAR data is represented in the dark blue color, and secondary LiDAR with light perano color. After line segmentation using the secondary LiDAR data, the remaining data is considered as a wall and is updated with the rest of the generated map.

**Figure 19:**
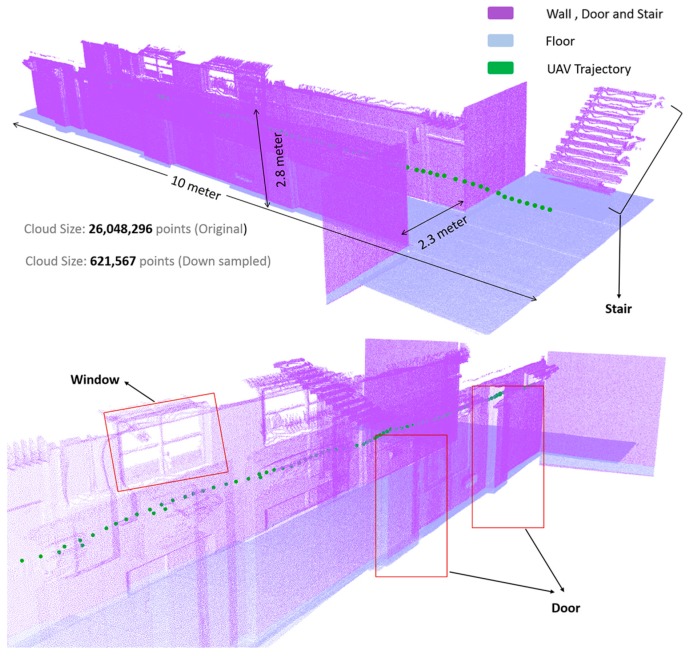
Generated indoor map and UAV path trajectory.

The mapping system was also evaluated using the navigation approach in order to construct an indoor environment outside the lab corridor. As shown in Figure 19, the generated map is sufficient for performing indoor modeling. For the experiment, we considered a straight corridor 15 m in length with some doors and pipeline structures. Even though the proposed approach can efficiently acquire the geometric information of indoor environment objects such as wall, door, and stairs, it is very difficult to capture the detailed geometry of objects such as spheres and cylinders. Therefore, laser scanned point cloud data from an industrial pipeline plant were virtually merged with the generated indoor map data for the UAV to detect and classify the pipelines.

### 5.3 Pipeline Classification

**Figure 20:**
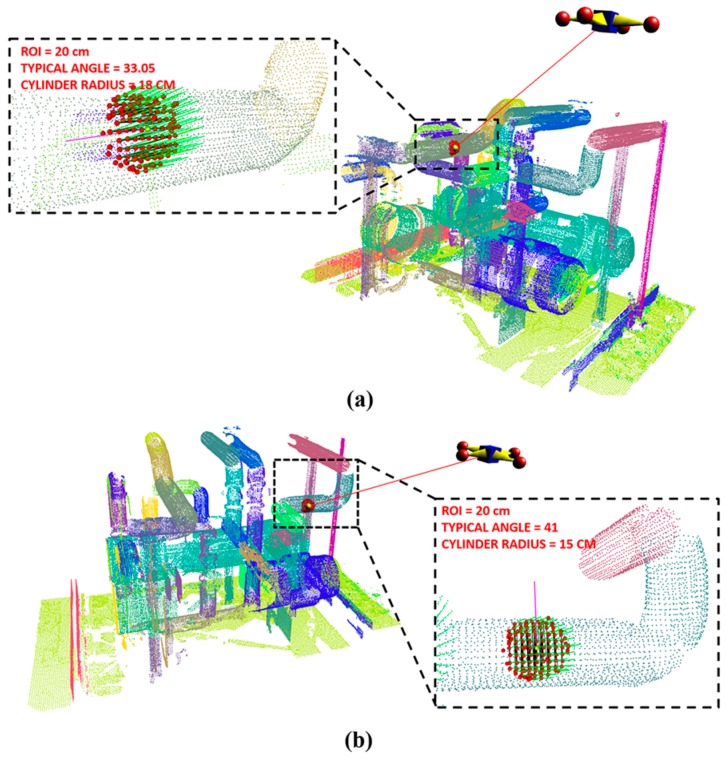
Industrial plant site laser scanned point cloud data: (**a**) 18 cm pipe and (**b**) 15 cm pipe.

The pipeline classification algorithm described in Section 3 was tested on the industrial plant site laser scanned point cloud data. The described method was implemented and tested in a C++ environment on a 3.07 GHz Intel Core i7 processor (Intel, Santa Clara, CA, USA) with 12 GB of memory. Figure 20 shows the industrial plant site at which the classification method was applied.

### 5.4. Comparision and Accuracy Evaluation

#### 5.4.1. Accuracy of Scan Matching

**Figure 21:**
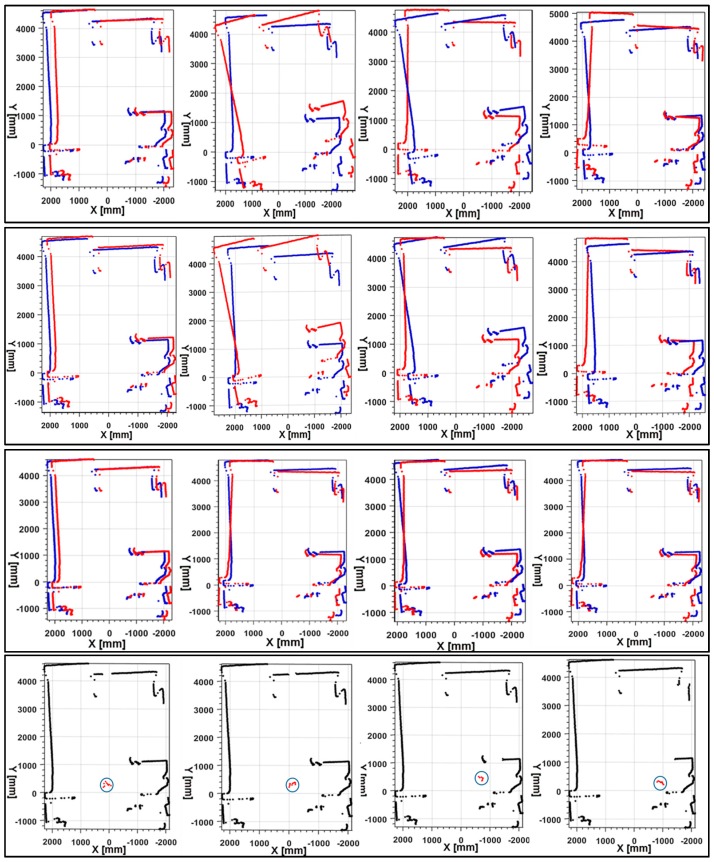
Scan matching results of 4 scan pairs using different methods. The first row is the results of ICP; the second row is the results of PSM; the third row is our scan matching results; the fourth is the ground truth results (Points with high intensity hilighted by cirlce). The plots in the first three rows describes the current scan S_t_ and reference scan S_t+1_ in blue and red dots, respectively.

The accuracy of the customized scan matching method was compared with other traditional scan matching ICP and PSM methods. The source codes of the PSM and ICP methods were obtained from the authors’ websites [19]. Figure 21 presents the visual matching results. The proposed method and other traditional methods were compared with 4 scan pairs selected randomly during the flight of a UAV. In this flight test, the UAV was assigned to fly at a constant altitude (3 m) with a predefined position, and we recorded the planar displacement within the test area using LiDAR (UTM-30LX-EW) intensity-based object tracking from a fixed position by attaching retro reflective tape to the UAV. The mean of the high intensity points was considered the position of the UAV.

Results of intensity-based object tracking are regarded as ground truth. As seen in the results, our method achieved similar results to the ground truth. Table 2 show the quantitative evaluation results of the consecutive scan data acquired from the LiDAR and with an integrated IMU. In the table, t_x_ and t_z_ represent the relative translation along the X-axis and Z-axis between the consecutive laser scan, respectively; θ represents the relative rotation angle between scan pairs. From Table 2, we can draw similar conclusions as in the visual comparison. As seen, our method estimated transformations were almost the same as the ground truth results. The maximum angular differences between our method and reference results were less than 0.03 rad (1.7188°). The maximum translation difference between the method and reference method was less than 3 cm. Even though the ICP and PSM methods had good success with stable platforms such as a mobile robot, they failed with UAV platforms. This failure occurs because, in both of the traditional methods, translation estimation works most accurately only if the correct orientation of the current scan is known. During the flight of the UAV, abrupt movement causes false estimation orientation of the LiDAR scan, producing results with inaccurate translation estimation. Thus, the proposed method is much more reliable even though the scanning platform is unstable.

#### 5.4.2. Computational Time

Computational time in the proposed navigation approach mainly depends on the scan matching and altitude estimation processes, in which time-consuming methods are unacceptable. The orientation estimation process is performed parallel with a separate processing unit that output at 80 Hz frequency is higher than the LiDAR measuring. In order to perform computational time analysis of the scan matching process and altitude estimation, we considered four scan pairs. Point density in each scan pair was 180, 360, 720 and 1080, respectively.

As seen in Table 3, determining the angular difference between two consecutive points from the integrated IMU reduces the computational time required by the other LiDAR standalone matching algorithms.

#### 5.4.3. Error Statistics Analysis

In Table 4, error distribution of the LiDAR scan matching and proposed LiDAR with IMU scan matching methods are compared. In this experiment, the scanning setup was kept in a stationary position in an indoor corridor for approximately 2 min. The results of scan matching from the two methods are compared to analyze the error distribution. In the stationary position, LiDAR scan matching was better than IMU integrated LiDAR matching. In a stationary position using the LiDAR scanning method, the incoming laser scan data showed no changes in consecutive scans and contained a very small amount of noise. However, when an IMU was integrated with LiDAR, the accumulated drift of the gyroscope and accelerometer undermined the accuracy of the final positioning result.

Another test was conducted to demonstrate the effectiveness of the LiDAR with integrated IMU scan matching method. In this test, the scanning platform was driven along the indoor corridor. The respective results of the IMU integrated LiDAR method were analyzed and compared. The results of error distribution for both methods in a dynamic position are shown in Table 5.

During dynamic position of the scanning platform, abrupt movement of the platform caused false orientation estimation with the LiDAR standalone method. These false measurements were inherent in the LiDAR scan matching method, but were eliminated by using IMU measurements. Hence, when the system was moving, the results of the LiDAR integrated IMU scan matching method were obviously better than those of the LiDAR standalone solution.

#### 5.4.4. Accuracy analysis for Pipeline Classification

**Figure 22:**
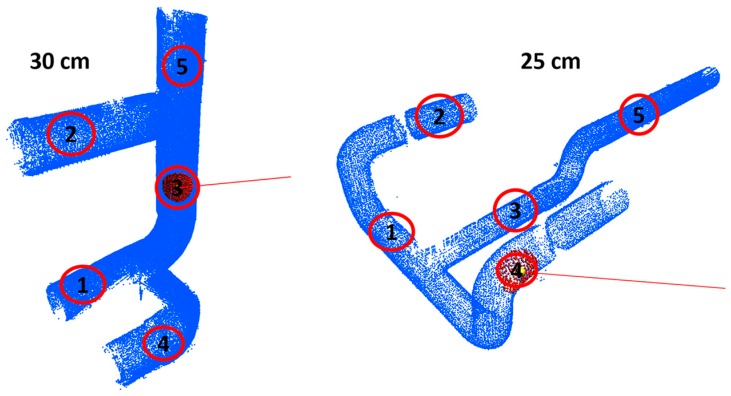
Radius estimation at random position on 30 cm and 25 cm cylinder point cloud data.

The accuracy of the proposed pipeline classification approach is measured by observing five random positions of ray casting on known 30 cm and 25 cm cylinder point cloud data [34], as shown in Figure 22. By considering five observation results (1–5), Table 6 shows that the accuracy analysis of the false rate (F_r_) of the proposed approach is lower and the precision rate (P_r_) is higher with increasing ROI.

## 6. Conclusions

In this paper, we propose a complete navigation solution for UAVs in an indoor environment by customizing and combining suitable existing algorithms. Also, the scan matching algorithm was extended to use with UAVs by stabilizing the LiDAR measurement plane using an IMU sensor. Innovations have been focusing on improving the algorithm efficiency and system integration with the use of inexpensive and lightweight range sensors. The main advantage of the system is the increased computational efficiency with the limited resources in the on-board computer. The experiment shows that the proposed navigation has significant potential for use in small UAVs in indoor mapping applications.

Extending the application area of the proposed navigation system, we proposed a new approach for classification of pipelines generated in an indoor map. The presented method utilizes the ROI and typical angle to estimate and classify the pipe radius. The ROI was enacted for estimating normal and the pairing of points since a wider or smaller ROI results in uncertainty when calculating point cloud features. The methodology was successfully tested on a synthetic pipeline as well as an industrial pipeline for real-world environmental point cloud data sets.

This work can continue in several directions. The navigation method can be improved by enhancing the scan matching algorithm with optimal computational complexity. Also, integrating a gyro-stabilized platform for LiDAR will improve the efficiency and robustness of the system by avoiding an ideal plane detection process. Thus, the limitation on the control of a UAV, especially during turns, can be removed completely. Additionally, the classification method can be extended to different indoor environment objects.

## Figures and Tables

**Table 1:** Typical angle range for pipeline classification with different ROIs.

ROI (cm)	Typical Angle Range			
	30 cm Pipe	25 cm Pipe	18 cm Pipe	15 cm Pipe
10	7°~10°	11°~13°	16°~28°	18°~25°
20	16°~19°	20°~25°	30°~38°	40°~54°
30	31°~37°	37°~41°	40°~59°	-

**Table 2:** Quantitative evaluation on scan data.

Scans	Scan Pair1			Scan Pair 2			Scan Pair 3			Scan Pair 4		
Method	LiDAR		IMU	LiDAR		IMU	LiDAR		IMU	LiDAR		IMU
	tx/cm	tz/cm	θ/rad	tx/cm	tz/cm	θ/rad	tx/cm	tz/cm	θ/rad	tx/cm	tz/cm	θ/rad
ICP	28.82	12.71	0.52	15.33	18.82	1.02	8.24	9.42	1.36	14.23	13.56	0.95
PSM	24.12	10.46	0.53	8.96	15.42	1.03	6.22	7.98	1.46	12.54	11.32	1.02
Proposed	16.48	7.32	0.48	9.51	10.43	1.01	3.88	8.97	1.49	11.03	7.58	0.97
Gound truth	18.60	6.83	0.5	12.12	13.11	1.0	5.96	11.04	1.5	10.24	8.95	1.0

**Table 3:** Computation time analysis.

Methods	180/ms	360/ms	720/ms	1080/ms
ICP	23	87	312	463
PSM	9	17	31	56
Proposed	2	5	11	16

**Table 4:** Stationary positioning error statistics.

Method	Data	RMS Error	Mean Error	Maximum Error
LiDAR	X	0.001	0.004	0.012
	Y	0.000	0.000	0.000
	Z	0.003	0.006	0.015
	Heading	0.000(deg)	0.000(deg)	0.000(deg)
IMU + LiDAR	X	0.004	0.018	0.024
	Y	0.000	0.000	0.000
	Z	0.013	0.018	0.027
	Heading	0.058(deg)	0.066(deg)	0.116(deg)

**Table 5:** Dynamic positioning error statistics.

Method	Data	RMS Error	Mean Error	Maximum Error
LiDAR	X	0.356	0.528	1.312
	Y	0	0	0
	Z	0.258	0.624	2.014
	Heading	0.124(deg)	0.523(deg)	0.339(deg)
IMU + LiDAR	X	0.008	0.010	0.016
	Y	0	0	0
	Z	0.0132	0.089	0.195
	Heading	0.023(deg)	0.015(deg)	0.042(deg)

**Table 6:** Accuracy analysis of pipeline classification for Figure 22.

ROI	G_R_	Measured Typical Angle/Radius					F_r_	P_r_
		1	2	3	4	5		
**10**	**30 cm**	8.13/30	6.54/NR	7.38/30	11.02/25	9.78/30	0.4	0.6
**20**		17.08/30	18.37/30	20.52/25	18.49/30	16.84/30	0.2	0.8
**30**		33.24/30	36.88/30	34.53/30	30.64/NR	32.67/30	0.2	0.8
**10**	**25 cm**	11.52/25	11.86/25	13.63/25	10.02/30	10.89/30	0.4	0.6
**20**		21.18/25	22.00/25	21.33/25	23.21/25	19.47/18	0.2	0.8
**30**		39.14/25	37.63/25	40.16/18	37.81/25	37.19/25	0.2	0.8

G_R_: Ground truth radius; F_r_: False rate; P_r_: Precision rate; NR: Not in Range.
